# Underwater Acoustic Sensors Based on Fiber Bragg Gratings

**DOI:** 10.3390/s90604446

**Published:** 2009-06-05

**Authors:** Stefania Campopiano, Antonello Cutolo, Andrea Cusano, Michele Giordano, Giuseppe Parente, Giuseppe Lanza, Armando Laudati

**Affiliations:** 1 Department for Technologies, University of Naples “Parthenope”, Centro Direzionale, Isola C4, 80143, Naples, Italy; 2 Department of Engineering, University of Sannio, Corso Garibaldi, 82100, Benevento, Italy; E-Mails: cutolo@unisannio.it (A.C.); a.cusano@unisannio.it (A.C.); 3 Istituto per i Materiali Compositi e Biomedici, CNR, 80125, Naples, Italy; E-Mail: gmichele@unina.it; 4 OptoSmart s.r.l., 80121, Naples, Italy; E-Mails: g.parente@optosmart.com (G.P.); g.lanza@optosmart.com (G.L.); a.laudati@optosmart.com (A.L.)

**Keywords:** fiber optics sensors, fiber Bragg gratings, hydrophones

## Abstract

We report on recent results obtained with a fiber optic hydrophone based on the intensity modulation of the laser light in a FBG (Fiber Bragg Grating) under the influence of the sound pressure. In order to control the behavior of the hydrophone in terms of sensitivity and bandwidth, FBGs have been coated with proper materials, characterized by different elastic modulus and shapes. In particular, new experiments have been carried out using a cylindrical geometry with two different coating, showing that the sensitivity is not influenced by the shape but by the transversal dimension and the material characteristics of the coating.

## 1. Introduction

In the last years, fiber Bragg grating (FBG) based sensors have been applied in a growing range of sensing applications, since their response to temperature or strain, or related measurands, is encoded as a linear or near-linear function of wavelength. Due to their extraordinary properties such as immunity to electromagnetic interference, remote sensing, stability in harsh environments, multiplexing capability, high sensitivity, wide dynamic range and simplicity FBG sensors offer solutions in situations where conventional sensors are unsuitable.

In particular, underwater acoustic measurement is an important area of interest, and a number of FBG pressure sensor configurations have been demonstrated [1-3]. The operating principle of a FBG-based hydrophone is typically based on the intensity modulation of the laser light due to the shift of transmission power spectrum curve of the sensing element under the influence of the acoustic field. However, for practical use, the low sensitivity to acoustic pressure of the FBG based sensors limit their use in underwater applications, where piezoelectric transducers are widely used, despite their dimensions, complex signal processing and electronic front-end and difficult multiplexing. This limit is essentially due to the high Young module of the optical fiber material (tens of GPa) which converts the effects of a high pressures applied on the grating in weak deformations.

Recently, the authors have shown how it is possible to enhance the sensitivity of FBGs in pressure and acoustic detection by coating the grating region with a material of low elastic modulus [4]. For a given acoustic pressure, the basic effect of the FBG coating is to enhance the dynamic strain experienced by the sensor of a factor given by the ratio between the fiber and the coating elastic modulus. According to this, an opportune coating can tailor the sensor directivity, the bandwidth and the acoustic sensitivity in water.

In this paper, the experimental characterization of several FBG based hydrophones coated with materials of different geometrical and acousto-mechanical properties are presented. In particular, starting from the results obtained in [4], new experiments have been carried out using a cylindrical geometry with two different coating: Damival and UR5041, showing that it is not the shape but the transversal dimension and the material characteristics of the coating that influence the sensitivity.

## 2. Operating Principle

The relation between the normalized Bragg wavelength shift Δλ_B_/λ_B_ and a spatial uniform sound pressure P(t) = p·sin(ω_S_t) around the FBG (where p and ω_S_ are the amplitude and angular frequency of the sound pressure, respectively), is given by [5]:

(1) $$\frac{\Delta \lambda_{B}}{\lambda_{B}}\left(t\right)=\left[-\frac{\left(1-2\nu \right)}{E}+\frac{n^{2}}{2}\frac{\left(1-2\nu \right)}{E}\left(2p_{12}+p_{11}\right)\right]P\left(t\right)$$

where n = 1.465, E = 70 GPa, ν = 0.17 and p_11_ = 0.121 and p_12_ = 0.270 are the effective refractive index of the guided mode, the Young's modulus, Poisson ratio and the elasto-optic coefficients of the optical fiber, respectively.

Thus, the spectral response of the FBG moves without changing its shape at the same frequency of the applied acoustic pressure. For a GE-doped FBG at 1550 nm, Δλ_B_/ΔP was measured as -3×10^-3^ nm/MPa over a pressure range of 70 MPa [6]. This means that with interrogation units able to perform wavelength shift measurements with a resolution of 10^-4^ pm in the investigated acoustic frequency range, an acoustic pressure limit of detection of hundreds of Pa can be obtained.

When optical fibers are coated with a plastic material, they exhibit some order of magnitude increase in their pressure sensitivity [7-8]. In fact, according to the Hocker analysis [9], if the FBG is coated with a thick layer of polymer, the normalized wavelength pressure sensitivity is given by:

(2) $$\frac{\Delta \lambda_{B}}{\lambda_{B}}\left(t\right)=\left[-1+\frac{n^{2}}{2}\left[p_{12}-\nu \left(p_{11}+p_{12}\right)\right]\right]\frac{\left(1-2\nu_{\text{coa}}\right)}{E_{\text{coa}}}P\left(t\right)$$

where E_coa_ and ν_coa_ are the Young's modulus and the Poisson ratio of the coating, respectively.

It can be seen from Equation 2 that for coatings with small Young's modulus compared with the fiber one, the wavelength pressure sensitivity of the FBG can be increased significantly. The experimental demonstration of the pressure gain sensitivity was proven in the static case [10], but the concept can be extended in the case of acoustic fields if the coating dimensions are small compared with the acoustic wavelength.

In addition, the extension of results is valid if the acoustic damping within the overlay is low in order to not affect the dynamic strain amplitude within the coating itself. Also, the acoustic impedance, related to the coating thickness and elastic modulus, should be very close to that of the water, in order to minimize the acoustic reflection at the water-coating interface.

## 3. Experimental Setup

The interrogation systems used to test the hydrophone is based on a stable narrowband wavelength tunable laser: its emitted optical signal wavelength has been fixed at the center of the linear region of the FBG's response; so, a translation of the FBG's spectral response, due to a incident acoustic wave, causes an amplitude variation of reflected optical power and then an output electric signal variation. The laser light intensity modulated by the FBG subjected to the sound pressure is detected by a fast photodiode.

Indeed, in the transmission mode and by working on the edge of the grating spectrum, the transmitted optical power P_t_ is directly related to the sound pressure according to the following expression:

(3) $$P_{t}\left(t\right)=P_{i}\left[T_{0}+\frac{\partial T}{\partial \lambda_{0}}\frac{\partial \lambda_{0}}{\partial p}P\left(t\right)\right]$$

where P_i_ is the incident optical power, T_0_ is the transmission value at FWHM, ∂T/∂λ_0_ and ∂λ_0_/∂p represent the edge slope of the grating spectral response and the wavelength sensitivity to the pressure, respectively. Yet, in order to achieve the maximum sensitivity and dynamic range, the laser wavelength should be set in correspondence of the full width at half maximum (FWHM), on either the longer or shorter wavelength side of the spectrum curve. It is seen from Equation 3 that the ac component of the transmitted light power is proportional to the sound pressure experienced by the FBG. Thus, the detection of the light with a photodiode provides an electrical output directly proportional to the acoustic field in the water. From the resulting temporal waveform, the amplitude, the frequency and the phase of the field can easily be measured after a proper calibration procedure.

**Figure 1.**
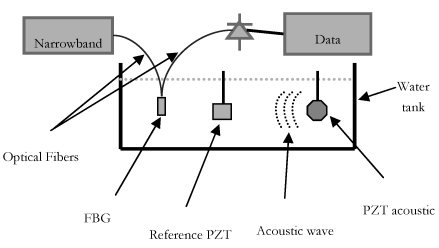
Lateral view of the experimental set-up.

The experimental set-up is shown in Figure 1. Field trials have been carried out in a professional tank at the Whitehead Alenia Sistemi Subacquei's laboratory. The tank size was 11 × 5 m, and its depth was 7 m. The sensor's symmetry axis was orthogonally to the direction of propagation of the acoustic wave. A piezoelectric hydrophone has been used as reference. A computer-controlled scanning stage that allows independent translations in X, Y and Z directions and rotations about the vertical axis is used to place the optical fiber hydrophone.

## 4. Results and Discussion

In this section, we report a series of measurements carried out to evaluate the sensitivity, the linearity and the resolution of the FBG-based hydrophone coated with materials of different geometrical and acousto-mechanical properties and to compare the obtained performances with a reference PZT hydrophone.

**Figure 2.**
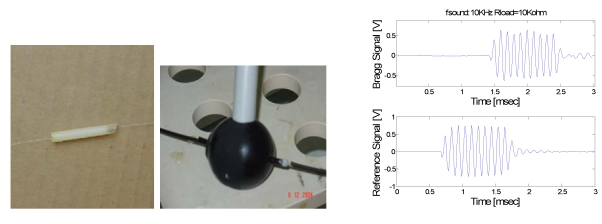
On the left: photographs of the tested hydrophones and, on the right, typical temporal response of the white coated FBG under test (upper) and of the reference hydrophone PZT (lower) to a sound pressure pulse of 2 kPa at the frequency of 10 kHz.

The first comparison is between two hydrophones with the FBGs (one characterized by a central wavelength of 1,554.20 nm and a FWHM of 0.5 nm and the other by a central wavelength of 1,547.6 nm and a FWHM of 0.45 nm) embedded in a material of cylindrical geometry with diameter of 4 mm and length of 25 mm exhibiting an elastic modulus of ∼100 MPa and in a material (the Damival 13650) of spherical geometry with diameter of 4.4 cm (exhibiting elastic modulus lower than 100 Mpa and acoustic impedance that matches that of water), respectively (see Figure 2).

The optical source was a stable narrowband wavelength tuneable laser and its output wavelength is tuned to the center of the slope of the transmission spectrum curve of the FBG under investigation. The power of light was set to a value of 6 mW. The laser light intensity modulated by the FBG subjected to the sound pressure is detected by a fast photodiode. Two optical isolators, one at the output of the laser, and another at the input of the photoreceiver, are inserted in order to stabilize the sensor output signal. The resolution of the system was estimated to be slightly less than 1 pm in the frequency range of interest. The outputs of the optical and piezoelectric (PZT) hydrophones were collected in a PC by an A/D acquisition system. The distance from the PZT transducer is of 1 m for the reference hydrophone PZT and 2.2 m for the FBG hydrophone, respectively.

Comparing the typical temporal response of the cylindrical-coated FBG hydrophone under test and the reference hydrophone PZT to a sound pressure pulse of 2 kPa at the frequency of 10 kHz, it can be seen that the FBG hydrophone operates as good as the piezoelectric technology. The phase difference between the two responses is due to the different distance from the acoustic source. Using the measured signal-to-noise ratio from the FFT of sensor response in Figure 2, the minimum detectable pressure level was estimated to be about 100 Pa.

**Figure 3.**
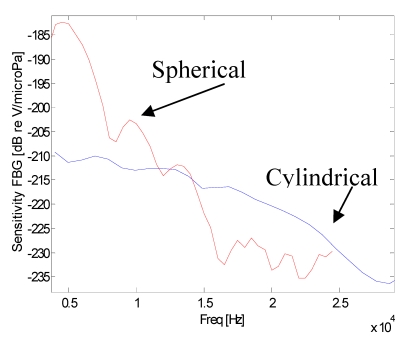
The calculated sensitivity curves of the FBG hydrophones.

In Figure 3, the calculated sensitivity curves of the FBG hydrophones are presented. The sensitivity curves (directly related to the Frequency Response) were obtained in two different ways: the first consists on the sensor interrogation with a train of sine-wave pulses of increasing frequency, the second consists on the sensor interrogation with a wideband pulse. In both cases, sensor sensitivities were estimated by using the piezoelectric data as reference. It can be seen from Figure 3 that the FBG hydrophones present a decreasing response in the range from low frequencies up to about 27 kHz and 16 kHz for the cylindrical and spherical coating, respectively. For upper frequencies the signal to noise ratio approaches the unity around the value of -235 dB reV/μPa. Furthermore, it can be seen from this figure that with respect to the cylindrical coating the spherical-coated hydrophone exhibits an higher sensitivity due to a better acoustic impedance matching with the water and to a smaller acoustic modulus whereas the smaller bandwidth is due to the wider dimensions. So, it is not the shape but the dimension and the material characteristics that influence the sensitivity. Obviously, under the same transversal dimension, the cylindrical coating is lighter, smaller, and easier to manage compared to the spherical coating. This is the reason why next experiments have been done using a cylindrical geometry and, as coating material, Damival and a new material, UR5041.

**Figure 4.**
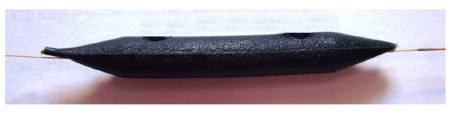
Photograph of the FBG hydrophones coated with UR5041.

In particular, taking into account also the results of the numerical simulations, it has been prepared a stamp, 30 mm long and with a diameter of 5 mm, and two other hydrophones with FBG embedded both in thermosetting polyurethane resins, Damival 13650 and UR5041, respectively. The polymers were cured at 60 °C for 24 h in the case of Damival 13650 and for 4 h in the case of UR5041. The choice of polyurethane resins is justified by their suitable elastic properties (Young's module about 100 MPa), low damping coefficient and excellent adhesion to fiber, features needed to get maximum transmission of acoustic waves and to minimize the absorption. A photograph of the hydrophone based on the UR5041 is presented in Figure 4.

**Figure 5.**
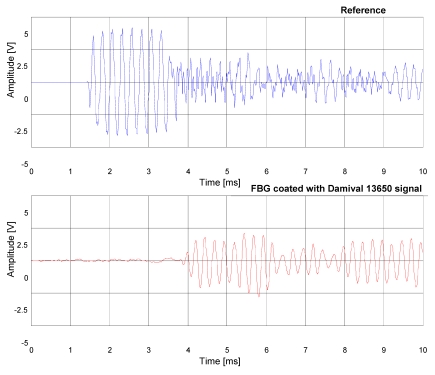
Temporal response of the reference hydrophone (upper) and the fiber optical hydrophone coated with Damival 13650 (lower) to a sound pressure pulse at the frequency of 4 kHz and duration of 2 ms.

**Figure 6.**
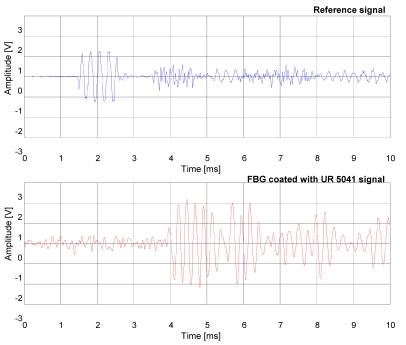
Temporal response of the reference hydrophone (upper) and the fiber optical hydrophone coated with UR5041 (lower) to a sound pressure pulse at the frequency of 4 kHz and duration of 1 ms.

Also in this case, the signal temporal response of the FBG hydrophone coated with Damival and the reference PZT hydrophone to a sound pressure pulse at the frequency of 4 kHz were compared (Figure 5). Furthermore, a comparison between the FBG hydrophone coated with the UR5041 and the reference hydrophone PZT (see Figure 6) have been carried out, where in order to increase the FBG hydrophone output voltage it has been used an higher gain of the electronic readout circuit. The distance from the PZT transducer is of 2.2 m for the reference hydrophone PZT and 6 m for the FBG hydrophones, respectively.

**Figure 7.**
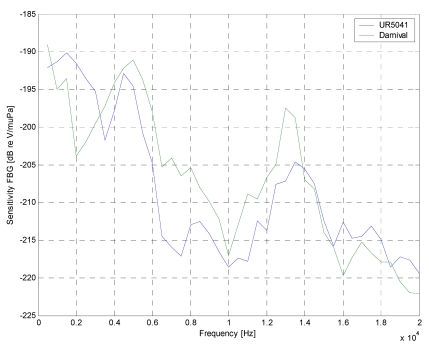
Frequency response of the FBG hydrophones coated with Damival and UR5041.

The frequency responses of the FBG hydrophones are shown in Figure 7. They were obtained varying the frequency of the pulse source in the range 500 Hz–20 kHz in 500 Hz steps.

It is noted that the spectral responses presented a frequency selective behavior: it had a maximum at the 4–6 kHz frequency range. This behavior wasn't due to fiber Bragg grating, which presented a wide and flat spectral response, but at the coating application; in particular, the frequency response depended on the material and on the geometry coating chosen. Also, the opto-acoustic response was limited by electronic front-end (passband filter with 3–5 kHz frequency range). The experimental results have demonstrated the capabilities of opto-acoustic antenna based on fiber Bragg gratings. It can get a fiber optic hydrophone with the same performances of the PZT traditionally hydrophone and with all advantages of the optical fiber sensors.

## 5. Conclusions

In conclusion, in this work, an innovative fiber-optical hydrophone based on polymeric coated FBG is reported, and it has been widely demonstrated the capability of such devices to measure vibrations and acoustic waves in the frequency range 3–5 kHz. The choice of the polymeric materials is winning both because it is possible to synthesize polymers with desired acousto-mechanical properties, and because their integration with the optical fibers result to be satisfactory. A FBG interrogation system based on a low cost detection scheme has developed and a maximum resolution of 10 Pa about was obtained. Work is in progress in order to enhance the sensor performance for pressure and acoustic measurements in a wide frequency range by coating the FBGs with new materials layers.
